# Analysis of the short-term effect of photodynamic therapy on primary bronchial lung cancer

**DOI:** 10.1007/s10103-020-03080-5

**Published:** 2020-06-27

**Authors:** Cunzhi Lin, Yuanyuan Zhang, Qian Zhao, Pingping Sun, Zhe Gao, Shichao Cui

**Affiliations:** Department of Respiration and Critical Care Medicine, The Affiliated Hospital of Qingdao University, Qingdao, 266000 Shandong China

**Keywords:** Photodynamic therapy, Bronchial lung cancer, Short-term clinical treatment effect, Clinical cases

## Abstract

To analyze the short-term clinical effect of photodynamic therapy on bronchial lung cancer and provide relevant practical experience for its better application in clinical practice. Twenty patients with bronchial lung cancer diagnosed by pathology were treated with photodynamic therapy or interventional tumor reduction combined with photodynamic therapy. Follow-up at 3 months after treatment, the chest CT and bronchoscopy were reexamined. The lesions were observed under a microscope, and the pathological specimens of living tissues were stained with HE and TUNEL to evaluate the short-term clinical effect. The volume of the tumor in the trachea or bronchus was smaller than before and the obstruction improved after the PDT from the chest CT. We could conclude that after PDT, the tumor volume was reduced and the pathological tissue appeared necrotic, the surface was pale, and the blood vessels were fewer while compared with before, and less likely to bleed when touched from the results of the bronchoscopy. HE staining showed that before treatment, there were a large number of tumor cells, closely arranged and disordered, or agglomerated and distributed unevenly. The cell morphology was not clear and the sizes were various with large and deeply stained nucleus, and the intercellular substance was less. After treatment, the number of tumor cells decreased significantly compared with before and the arrangement was relatively loose and orderly. The cells were roughly the same size; the intercellular substance increased obviously and showed uniform staining. The nuclei morphology was incomplete and fragmented, and tumor cells were evenly distributed among the intercellular substance. TUNEL staining showed that the number of cells was large and the nucleus morphology was regular before treatment; the nuclear membrane was clear and only a small number of apoptotic cells could be seen. However, the number of cells decreased and arranged loosely after treatment, with evenly stained cytoplasm. The nuclear morphology was irregular and the nuclear membrane cannot be seen clearly. Apoptotic cells with typical characteristics such as karyopyknosis, karyorrhexis, and karyolysis were common. Photodynamic therapy for bronchial lung cancer can achieve a satisfactory short-term clinical treatment effect and improve the life quality of patients, but the long-term clinical effect remains to be further studied.

## Introduction

Lung cancer is one of the malignant tumors with the highest morbidity and mortality around the world, and its incidence is increasing year by year, which is seriously endangering human health [1, 2]. Early patients can be treated with surgery, but some patients with bronchial lung cancer often have missed the opportunity of radical surgical resection when they are diagnosed. Chemotherapy and radiotherapy are greatly affected by the patient’s physical conditions, and the side effects are serious, while bio-targeted therapy is not necessarily suitable for everyone and is easy to produce drug resistance. As a minimally invasive treatment for malignant tumors, photodynamic therapy (PDT) can significantly improve the prognosis of patients with bronchial lung cancer and improve their quality of life [3]. The term “photodynamic therapy” was first coined by Tapperner in 1907 [4] and gradually developed in the late 1970s for the treatment of tumors. However, until 1978, Dougherty et al. used PDT to conduct systematic treatment research on skin and subcutaneous tumors [5]. As one of the photosensitizers approved for use in China, hematoporphyrin derivatives (HPDs) are mainly used for the treatment of tumors [6]. In 1960, Lipson et al. developed the photodynamic characteristics of HPD, which were clinically applicable to breast cancer patients in 1966. It has been proved that after several hours, injection of HPD performed PDT can selectively destroy metastatic breast cancer. In 1996, hematoporphyrin became the first photosensitizer officially approved for clinical use. As the representative of the first-generation photosensitizer, it is widely used in the treatment of various diseases. In recent years, with the development of semiconductor lasers and new photosensitizer, PDT has been paid more and more attention in the treatment of tumors and has become one of the most active research fields in the prevention and treatment of cancer in the world.

However, there is no worldwide consensus and treatment method is mainly based on previous experience. In this paper, the prognosis and symptom remission of patients with bronchial lung cancer treated by PDT in our hospital were studied to analyze the short-term clinical effect of PDT on bronchial lung cancer and to provide more practical experience for its better application in clinical practice.

## Materials and methods

### Clinical data

Twenty patients diagnosed with bronchial lung cancer in Huangdao Hospital District, The Affiliated Hospital of Qingdao University were collected. Inclusion criteria: (1) Pathological diagnosis was lung adenocarcinoma, squamous cell carcinoma, or mucinous adenocarcinoma; (2) the treatment method was PDT or combined PDT after interventional tumor reduction. Exclusion criteria: (1) Simple surgical resection without PDT; (2) those whose physical condition were unable to tolerate the bronchoscope.

Among the 20 patients, there were 15 males and 5 females. The patients were aged (66.8 ± 7.30) years, with a median age of 66.5 years. There were 16 cases of squamous cell carcinoma, three of which were squamous cell carcinoma with mucinous adenocarcinoma and 4 cases of adenocarcinoma (Table 1). Thirteen cases were treated with combined PDT after interventional tumor reduction, and 7 cases were treated with PDT without tumor resection. All patients were both treated combined with chemotherapy or radiation or targeted therapy. Twenty patients were diagnosed as bronchial lung cancer by chest CT, bronchoscopy, percutaneous lung biopsy, and histopathology. The informed consent was signed by the patients and their families, and this study was approved by the hospital ethics committee.

**Table 1:** The demographics, smoking status, and clinical characteristics of patients

Cases	Gender (male: M, female: F)	Age (years)	Smoking index	Pathologic types (squamous cell carcinoma: S, adenocarcinoma: A)	Clinical stage (TNM)
1	M	48	1200	S	T4N2M0 (IIIB)
2	M	66	2000	S	T4N2M0 (IIIB)
3	M	73	800	S	T4N2M0 (IIIB)
4	F	66	No	S	T4N0M0 (IIIA)
5	M	67	1500	S	T4N0M0 (IIIA)
6	M	73	1600	S	T4NOM0 (IIIA)
7	F	68	No	A	T2aN3M1b (IVA)
8	M	65	1200	S+A	T4N3M1a (IVA)
9	M	66	800	S+A	T4N4MO (IIIC)
10	F	66	No	A	T4N0M1a (IVA)
11	M	77	No	S	T4N2M0 (IIIB)
12	M	68	No	S	T2N2M1c (IVB)
13	M	71	No	A	T4N0M0 (IIIA)
14	M	69	No	A	T4N4MO (IIIC)
15	M	78	No	S	T4NOM0 (IIIA)
16	M	56	No	S	T4NOM1 (IIIA)
17	F	54	No	S	T4N2M0 (IIIB)
18	F	66	No	S	T4N0M0 (IIIA)
19	M	73	1600	S	T4NOM0 (IIIA)
20	M	66	800	S+A	T4N4MO (IIIC)

### Photodynamic therapy

Indications: (1) Indications for radical treatment: Early primary central lung cancer, no lymph node metastasis, and distant metastasis were found in imaging examination; the maximum diameter of unilateral lesion was less than 1 cm in those who were unable or refused surgical operation; the precancerous lesion of trachea and bronchus only involved mucous membrane and submucosa, and the length and infiltration depth were both less than 1 cm. (2) Palliative treatment: The maximum diameter of primary or metastatic unilateral lesion was more than 2 cm and blocked the lumen, causing dyspnea; local recurrence of stump after operation of lung cancer; refractory endobronchial neoplasms that recurred after radiotherapy and chemotherapy and blocked the tracheobronchial lumen; the lesion invaded the bronchial cartilage or the outer membrane.

Contraindications: Allergic to photosensitizers; hematoporphyrin and other diseases worsened by light, such as systemic lupus erythematosus and dermatomyositis; obvious coagulation dysfunction; patients with severe cardiopulmonary insufficiency, hepatorenal insufficiency, hypertension, history of heart disease; pregnant women; the tumor has invaded the peripheral large blood vessels. Destruction of the tracheal wall; the patient was in a state of cachexia and the estimated survival time is less than 3 months.

## Methods

Photodynamic therapy is based on the guidelines of “Chinese expert consensus on Clinical Application of Photodynamic Therapy for Respiratory tract tumors.” Before PDT, patients and their families should be informed of the process of PDT, intraoperative risks and possible postoperative complications, prognosis, and follow-up, and the advantages and disadvantages of PDT should be explained in detail and carried out with the consent of patients and family members. In addition, it is necessary to perfect relevant inspection, including blood routine examination, liver and kidney function, blood coagulation function, infectious markers (hepatitis B virus, hepatitis C virus, human immunodeficiency virus, syphilis), pulmonary function test, electrocardiogram, chest CT, and fiberoptic bronchoscope. It is to determine whether the patients can tolerate photodynamic therapy under bronchoscope and to identify the location and extent of the lesion, degree of lumen obstruction, etc. Skin test should be carried out before the injection of photosensitizer, and the local reaction should be observed 15 min later. Only those who are negative can use the drug. Patients were given intravenous injection of hematoporphyrin injection Hiporfin (Chongqing Milelonge Biopharmaceutical Co. Ltd) 2.5 mg/kg. Strictly avoid light and direct sunlight within 1 week after the injection, the patients should avoid light and direct sunlight strictly so patients can stay in a dark room equipped with shade curtains and lighting less than 60 watts, wear sunglasses, and forbid playing with mobile phones or computers. According to the length and range of the lesion, the optical fibers (Guilin Xingda Photoelectric Medical Equipment Co. Ltd) with different dispersion segment length (2~6 cm) were selected and introduced into the lesion through the bronchoscope. The lesion was irradiated by semiconductor laser with a wavelength of 630 nm routinely. The power density was 100 mW/cm^2^ and the total energy density was 100~150 J/cm^2^, then the irradiation time was calculated. Segmentalized irradiation and intermittent laser irradiation should be chosen if the diffuse lesion and tumor basal area is relatively wide, which is conducive to the recovery of tissue oxygen concentration and improve the curative effect. In general, 48 h after administration, when the drug concentration difference between the tumor tissue and the surrounding normal tissue reached optimal, the first laser irradiation was performed. The second laser irradiation was performed 72 h later; it is necessary to clean up the surface necrotic material moderately caused by the first treatment, and avoid excessive bleeding. The irradiation energy should be determined according to the size of the tumor, and should not exceed the energy that of the first irradiation. The necrotic material should be cleaned in time during and 1 week after photodynamic treatment to avoid dyspnea caused by obstruction of the lumen. Local anesthesia or general anesthesia was selected after evaluating the operation time according to the patient’s physical condition, tumor location, and size. For patients with large tumor that cause severe obstruction of the trachea and bronchus, the residual roots could be treated with photodynamic therapy after resection, argon knife cauterization, or electric snare ligation of tumor under general anesthesia, which could obtain better efficacy. After the completion of PDT, patients were instructed to continue to avoid light, the treatment of possible complications and other matters needing attention. If the patients had common minor complications such as fever, hemoptysis, chest tightness, and photosensitivity, it can be symptomatic treated routinely; if the patients had serious complications such as dyspnea or even asphyxia caused by acute mucosal edema, perforation, or fatal massive hemoptysis, they should seek medical advice in time for endotracheal intubation or tracheotomy to save lives. After 3 months, the chest CT and fiberoptic bronchoscope were reexamined to judge the curative effect, and pathological samples were taken for staining. If there were residual lesions, photodynamic therapy could be performed again according to the situation.

## Criteria for evaluating clinical efficacy

Chest CT and bronchoscopy were reexamined 3 months after PDT treatment, and the curative effect was evaluated from the result of chest CT, bronchoscopy, the pathological tissue hematoxylin-eosin (HE) staining, TdT-mediated dUTP nick end labeling (TUNEL) staining, and symptom relief. The results are shown as follows.

## Results

### Chest CT results

**Fig. 1:**
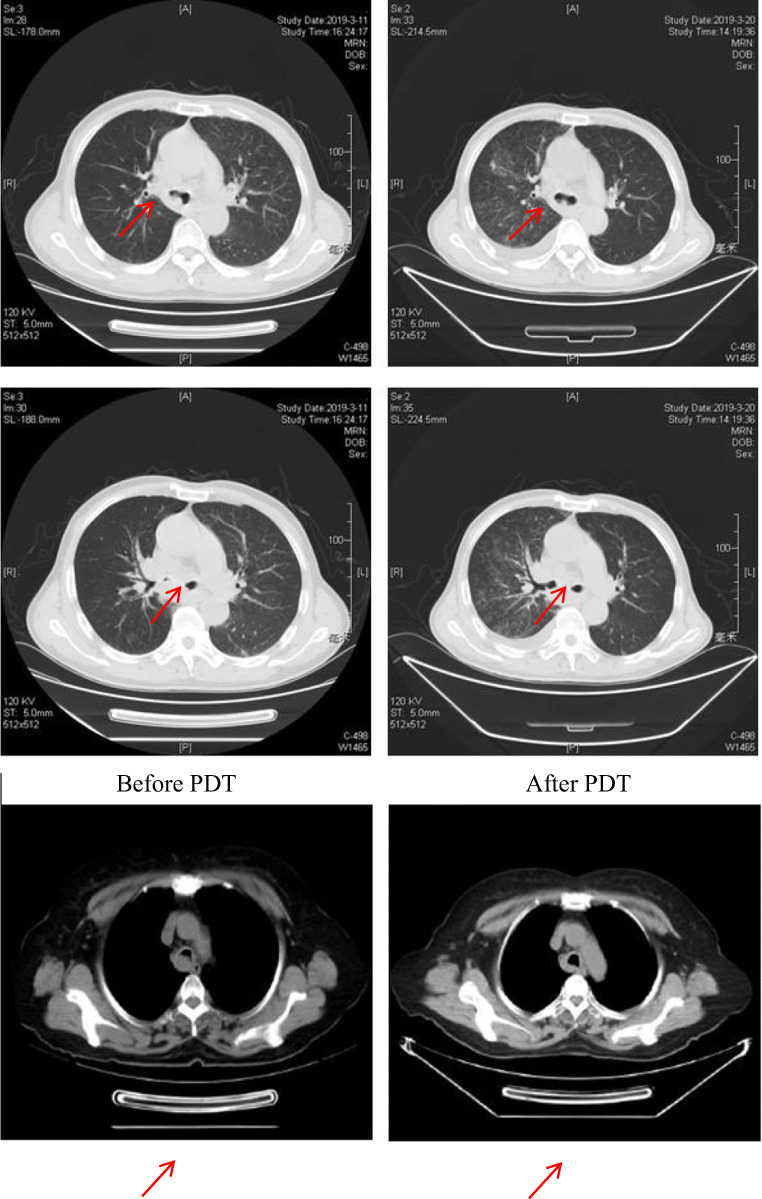
Changes in chest CT 3 months later after PDT. The first column was before the photodynamic treatment, and the second column was the corresponding after treatment

As could be seen from the chest CT, the volume of the tumor in the trachea or bronchus was smaller than before and the obstruction improved after the PDT (Fig. 1).

### Results of bronchoscopy

**Fig. 2:**
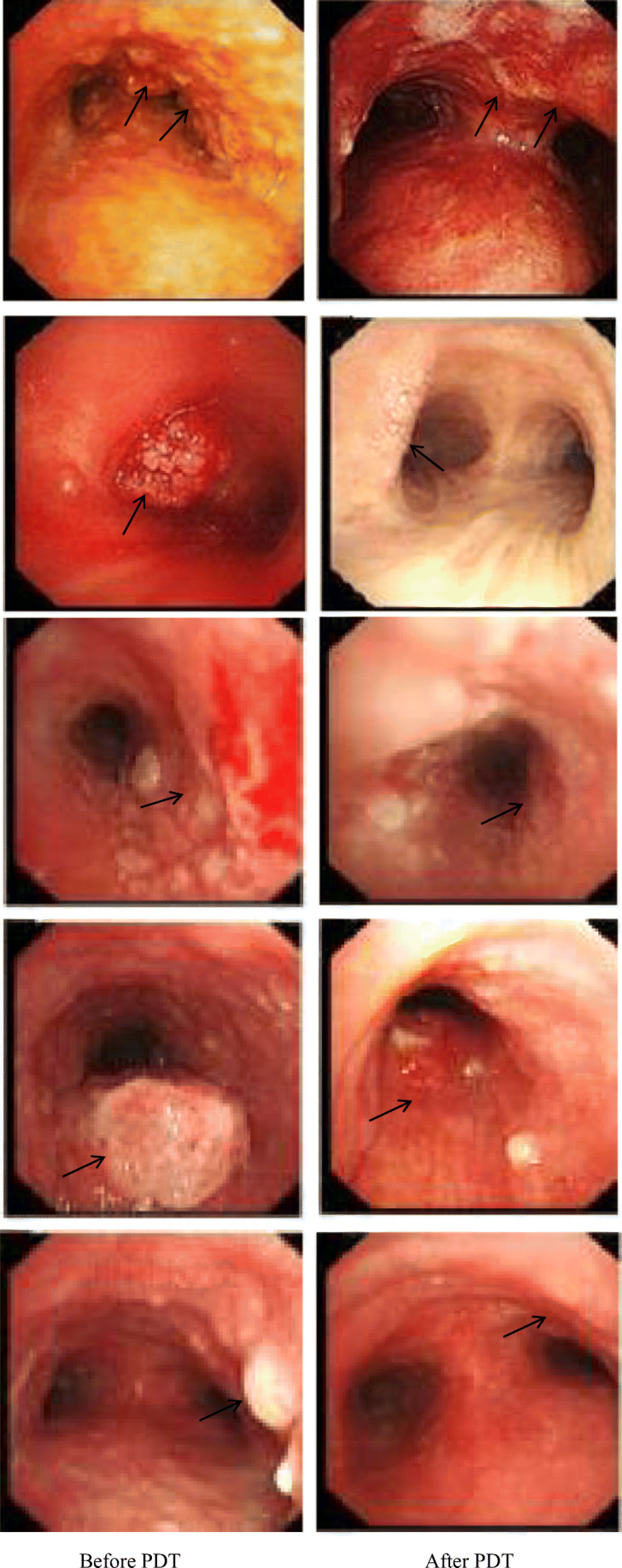
Changes in bronchoscopy 3 months later after PDT. The first column was before the photodynamic treatment, and the second column was the corresponding after treatment

From the results of the bronchoscopy, we could conclude that after PDT, the tumor volume was reduced and the pathological tissue appeared necrotic, the surface was pale, and the blood vessels were fewer while compared with before, and less likely to bleed when touched (Fig. 2).

### Pathological HE staining results

**Fig. 3:**
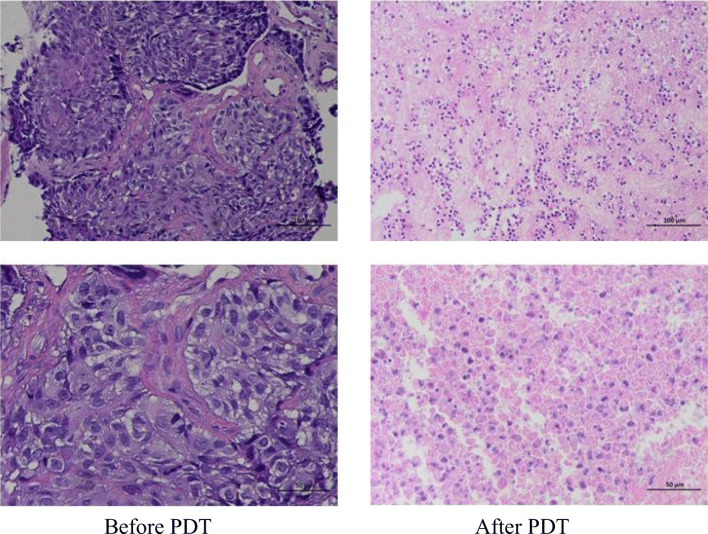
Changes in the pathological tissue HE staining 3 months later after PDT. The first column was before the photodynamic treatment, and the second column was the corresponding after treatment

HE staining showed that before treatment, there were a large number of tumor cells, closely arranged and disordered, or agglomerated and distributed unevenly. The cell morphology was not clear and the sizes were various with large and deeply stained nucleus, and the intercellular substance was less. After treatment, the number of tumor cells decreased significantly compared with before and the arrangement was relatively loose and orderly. The cells were roughly the same size; the intercellular substance increased obviously and showed uniform staining. The nuclei morphology was incomplete and fragmented, and tumor cells were evenly distributed among the intercellular substance (Fig. 3).

### Pathological TUNEL staining results

**Fig. 4:**
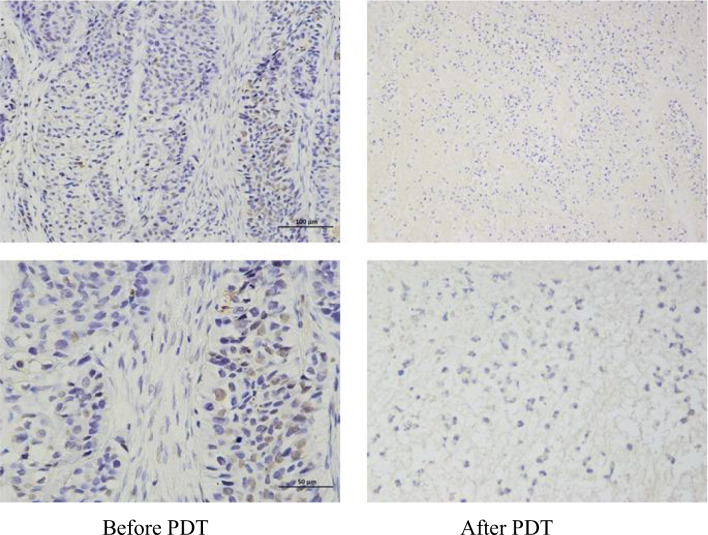
Changes in the pathological tissue TUNEL staining 3 months later after PDT. The first column was before the photodynamic treatment, and the second column was the corresponding after treatment

TUNEL staining showed that the number of cells was large and the nucleus morphology was regular before treatment; the nuclear membrane was clear and only a small number of apoptotic cells could be seen. However, the number of cells decreased and arranged loosely after treatment, with evenly stained cytoplasm. The nuclear morphology was irregular and the nuclear membrane cannot be seen clearly. Apoptotic cells with typical characteristics such as karyopyknosis, karyorrhexis, and karyolysis were common (Fig. 4).

### Symptom remission results

Bronchial obstruction was better than before; the patient complained of relief of dyspnea and cough was alleviated, occasionally hemoptysis and necrotic material. The favorable and unfavorable outcomes in patients in the two types of offered therapy PDT alone and PDT with surgery were as follows (Table 2). From the result of chest CT, bronchoscopy, the pathological tissue HE staining, TUNEL staining, and symptom relief, we could conclude that PDT combined with surgery was more effective than PDT alone.

**Table 2:** Favorable and unfavorable outcomes in patients in the PDT alone and PDT with surgery

	PDT alone	PDT with surgery
Favorable outcome	It takes less time so no anesthesia is needed. Less invasive to the body bronchial lumen and less likely to bleed.	The tumor tissue in the lumen was surgical resected first to reduce tumor load, and then laser irradiation was used to increase the degree of tumor necrosis and apoptosis. Less likely to relapse than PDT alone and is more effective than PDT alone.
Unfavorable outcome	PDT alone is only limited to tumor tissues without bulge in the lumen, that is, diffuse invasion and unresectable tumor tissues.	It takes a long time and the patient needs general anesthesia. During the resection of the tumor, it is very prone to bleeding and even perforation

## Discussion

The common treatments for lung cancer include surgery, radiotherapy, chemotherapy, and targeted therapy. In recent years, with the development of interventional medicine, PDT, as a new minimally invasive method for tumor treatment, has gradually attracted researchers’ attention and been increasingly applied in the clinical treatment of respiratory malignancies. PDT is particularly useful for patients with advanced disease and those with early central lung cancer but are unable to undergo surgery [7–9]. It also has good compatibility with common treatment methods [3]. It can be combined with interventional tumor reduction under bronchoscope or surgery to achieve better results [10]; In addition, studies have confirmed that PDT combined with radiotherapy can produce superimposed therapeutic effect, which is safe and effective [11, 12]; Moreover, PDT combined with chemotherapy is not only effective but also can reduce the number of chemotherapy cycle, thus reducing the side effects on the body. [13–15]. Combined with molecular-targeted drugs can not only improve its drug resistance but also enhance the effect of photodynamic therapy [16]. However, the researches of PDT combined with immunotherapy are still in the laboratory stage, and the curative effect of its combination is unclear, which needs to be further studied.

PDT is a minimally invasive therapeutic method with low toxicity [3], repeatability [17], specificity, and applicability. Photodynamic therapy consists of three essential components that is photosensitizer, light, and oxygen [18]. It makes use of the high affinity of the photosensitizer to the tumor tissue, so that the photosensitizer accumulates in the tumor tissue, and when the concentration difference of the photosensitizer between the tumor tissue and the surrounding normal tissue reached optimal, appropriate wavelength of laser is used to irradiate the diseased tissue to stimulate the photosensitizer, which absorbs energy and transitions to the excited state. When it returns to the ground state, it can release energy and transfers it to molecular oxygen then produce toxic photochemical product–oxidizing active substances such as free radicals and singlet oxygen (^1^O_2_), which produce a series of biological effects through various mechanisms that eventually lead to the apoptosis, necrosis, or autophagy of tumor cells [3, 19–22]. Furthermore, PDT can damage the vascular endothelium of tumor tissue and cause thrombosis, inhibit the formation of neovascularization thus blocking the blood supply of tumor, resulting in tissue ischemia and hypoxia [23, 24]. PDT can also activate the anti-tumor effect of the body’s immune system and induce the formation of a variety of immune molecules and to eliminate tumor cells, which is of great significance in preventing tumor recurrence [25–29]. The selective accumulation of photosensitizers in tumor tissues and the selective laser irradiation on the pathological tissues together constitute the double targeting effects of PDT (drug-targeted aggregation and laser-targeted activation) [30]. Photosensitizers accumulate in significantly higher concentrations in cancer cells than in regular cells [31]; therefore, the damage to normal tissues is slight and easy to recover.

In this study, the short-term clinical efficacy of PDT for bronchial lung cancer was evaluated by analyzing the result of chest CT, fiberoptic bronchoscope, the pathological tissue HE, TUNEL staining, and symptom relief 3 months later after PDT or interventional tumor reduction combined with PDT. The results showed that the tumor was smaller than before and the obstruction of trachea or bronchus was improved concluded from chest CT. Fiberoptic bronchoscopy revealed that the surface of the lesion was gray or necrotic material was fell off and coughed out. HE staining showed that before treatment, there were a large number of tumor cells, closely arranged and disordered, or agglomerated and distributed unevenly. The cell morphology was not clear and the sizes were various with large and deeply stained nucleus, and the intercellular substance was less. After treatment, the number of tumor cells decreased significantly compared with before and the arrangement was relatively loose and orderly. The cells were roughly the same size; the intercellular substance increased obviously and showed uniform staining. The nuclei morphology was incomplete and fragmented, and tumor cells were evenly distributed among the intercellular substance. TUNEL staining showed that the number of cells was large and the nucleus morphology was regular before treatment; the nuclear membrane was clear and only a small number of apoptotic cells could be seen. However, the number of cells decreased and arranged loosely after treatment, with evenly stained cytoplasm. The nuclear morphology was irregular and the nuclear membrane cannot be seen clearly. Apoptotic cells with typical characteristics such as karyopyknosis, karyorrhexis, and karyolysis were common. The result of symptom remission revealed that bronchial obstruction was better than before; the patient complained of relief of dyspnea and cough was alleviated, occasionally hemoptysis and necrotic material. Moreover, PDT combined with surgery was more effective than PDT alone.

To sum up, PDT for bronchial lung cancer can achieve a satisfactory short-term clinical treatment effect and improve the life quality of patients, but the long-term clinical effect remains to be further studied.
